# Supplementary figures and images for: Embryonic Valproate Exposure Alters Mesencephalic Dopaminergic Neurons Distribution and Septal Dopaminergic Gene Expression in Domestic Chicks
Source: Front Integr Neurosci. 2022 Mar 16;16:804881. doi: 10.3389/fnint.2022.804881 (PMC8966611; doi:10.3389/fnint.2022.804881)

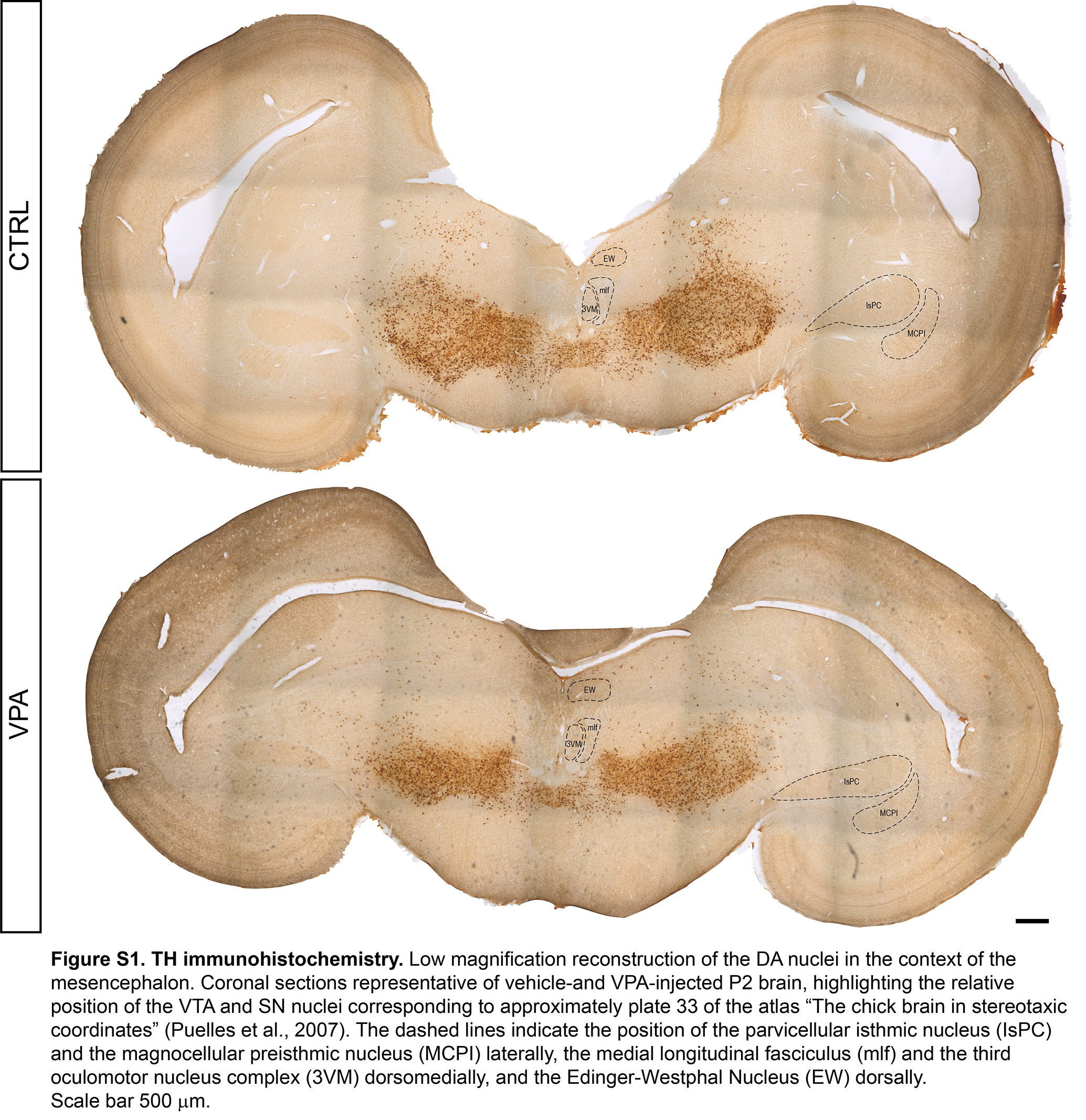

Supplement: Supplementary file 2 [file Image_1.jpg]
